# Proanthocyanidin associated to vitamin E or palm oil on initial enamel erosion: *in situ* and *in vitro* study

**DOI:** 10.1590/1807-3107bor-2025.vol39.081

**Published:** 2025-09-08

**Authors:** Daiana da Silva MARTINS, Ana Paula BOTEON, Thayná Teodoro da SILVA, Julia Fiorese SABINO, Franciny Querobim IONTA, Angélica Aparecida de OLIVEIRA, Heitor Marques HONÓRIO, Marília Afonso Rabelo BUZALAF, Thiago Saads CARVALHO, Daniela RIOS

**Affiliations:** (a) Universidade de São Paulo – USP, Bauru School of Dentistry, Department of Pediatric Dentistry, Orthodontics and Public Health, Bauru, SP, Brazil.; (b) Universidade de São Paulo – USP, Ribeirão Preto School of Dentistry, Department of Children’s Clinic, Ribeirão Preto, SP, Brazil.; (c) Universidade de São Paulo – USP, Bauru School of Dentistry, Department of Biological Sciences, Bauru, SP, Brazil.; (d) University of Bern, Department of Preventive, Restorative and Pediatric Dentistry, Bern, Switzerland.

**Keywords:** Vitamin E, Vitis, Palm Oil, Dental Pellicle, Tooth Erosion

## Abstract

This *in vitro* study evaluated the effect of proanthocyanidin, palm oil, and vitamin E against initial erosion. Bovine enamel blocks (n = 140) were divided into 14 groups: C+_SnCl2/NaF/Am-F-containing solution (positive control); C-_deionized water (negative control); O_palm oil; P6.5_6.5% proanthocyanidin; P2_2% proanthocyanidin; E_Vitamin E (97.8% oily tocopherol acetate); OP6.5_palm oil + 6.5% proanthocyanidin; P6.5O_6.5% proanthocyanidin + palm oil; OP2_palm oil + 2% proanthocyanidin; P2O_2% proanthocyanidin + palm oil; EP6.5_Vitamin E + 6.5% proanthocyanidin; P6.5E_6.5% proanthocyanidin + Vitamin E; EP2_Vitamin E + 2% proanthocyanidin; P2E_2% proanthocyanidin + Vitamin E. The acquired enamel pellicle (AEP) was previously formed *in situ* for 30 min. The specimens were treated *in vitro* with the solutions (500 µL, 30s). Then, the blocks were maintained for an additional hour in oral cavity to develop the modified AEP. The blocks were immersed in 0.5% citric acid (pH 2.5) during 30s. The response variable was the percentage of surface hardness loss (%SHL). Data were analyzed by one-way ANOVA and Fisher’s LSD test (p<0.05). P6.5E (12±7_%SHL) was the only group that promoted similar protection to C+ (11±8_%SHL). O (17±13_%SHL), P2 (20±10_%SHL), OP6.5 (19±12_%SHL), P2O (21±13_%SHL), P6.5E (12±7_%SHL), and P2E (19±9_%SHL) exhibited %SHL similar to both C+ and C- (25±10_%SHL) groups (p<0.05). P6.5 (23±11_%SHL), E (27±8_%SHL), P6.5O (24±13_%SHL), OP2 (27±12_%SHL), EP6.5 (24±11_%SHL), and EP2 (26±11_%SHL) were different to C+ and similar to C-. It was concluded that the combination of 6.5% proanthocyanidin and vitamin E (P6.5E) was the most effective strategy against enamel erosion, aligning closely with the positive control.

## Introduction

Dental erosion refers to the process of chemical softening of the tooth surface subjected to acid exposure.^1^ When mechanical forces are associated (abrasion or attrition, for example), the process advances causing irreversible structural tissue loss. At this stage, the phenomenon is termed erosive tooth wear and has the potential to adversely impact individuals’ quality of life^1^ by causing issues like pain, sensitivity, loss of function, and compromised aesthetics, thereby underscoring the critical importance of controlling the progression of this condition.^1^ Therefore, to prevent these events, it is necessary to manage the causal factors of erosive tooth wear, such as changing eating and oral hygiene habits, treating gastric and general health conditions and other functional problems.^2^

Saliva contributes greatly to the control of dental erosion by forming an acquired pellicle that protects the enamel surface from immediate contact with acidic agents, slowing down the enamel demineralization.^3,4^ Nevertheless, in the face of severe erosive challenges, the acquired enamel pellicle (AEP) proves insufficient to mitigate the impact of acids on the tooth surface.^5^ Lipids constitute 25% of the composition of the AEP and may play an important role in its protective effect on enamel against erosive acids.^6-8^ In previous studies, palm oil has shown efficacy in preventing enamel erosion,^6-8^ with its presumed mechanism attributed to a composition rich in tocotrienols.^9^ These tocotrienols are believed to diffuse through the AEP, consequently retarding its disintegration.^6,7^ Despite its low cost and easy accessibility, palm oil has certain unfavourable characteristics, such as unpleasant taste and potential for staining, which could render its application in oral hygiene products impractical. A previous study found that the specific component responsible for such protection is vitamin E.^8^

Another agent with notable efficacy in protecting against dental erosion is proanthocyanidin,^10,11^ a polyphenol derived from fruits, vegetables, peels, and seeds, predominantly sourced from grape seeds.^23^ Proanthocyanidin can form insoluble complexes with carbohydrates and proteins and interact with salivary proteins, such as proline-rich proteins (PRPs), primarily through hydrogen bonding.^13,14^ This results in the enhancement of AEP’s^13,14^ preventive effect against dental erosion.^11^ Given the preventive mechanisms of palm oil, its active agent vitamin E, and proanthocyanidin, it would also be intriguing to explore whether their combination with this polyphenol enhances their protective effects in preventing erosion. This conceptual framework is based on the idea that palm oil or vitamin E may facilitate the dispersion of proanthocyanidin within the AEP, potentially augmenting its protective capacity. Thus, the objective of this study was to evaluate the effect of proanthocyanidin combined with either palm oil or vitamin E, as well as the individual effects of these agents, against initial enamel erosion *in vitro*. The formulated null hypotheses are as follows: (H1) palm oil, vitamin E, and proanthocyanidin do not provide protection against enamel erosion; (H2) the interaction of proanthocyanidin with palm oil or vitamin E do not result in protection against enamel erosion.

## Methods

### Experimental design

This study was conducted according to the Declaration of Helsinki. The protocol was approved by the local Research Ethics Committee (Protocol 57816322.7.0000.5417). All individuals signed an informed consent form before the confirmation of their eligibility for the study.

**Figure 1:**
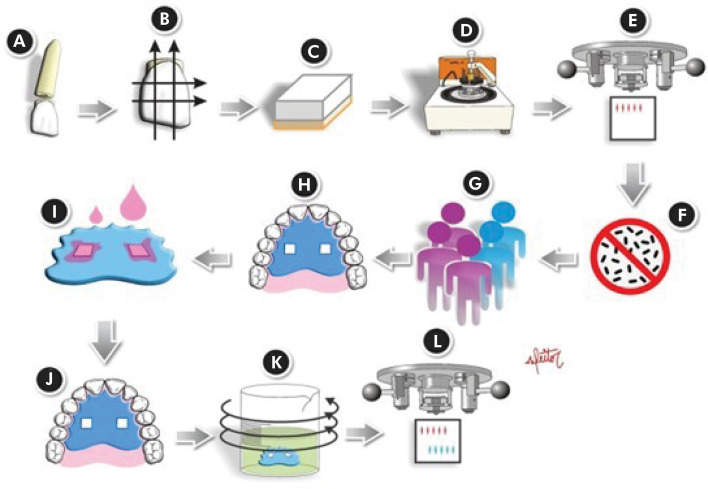
Experimental design of the study. A. Separation of the crown and root of the bovine tooth. B. Demarcation of the region of the crown to be cut. C. Cut specimen (4x4 mm). D. Polishing of specimens. E. Initial surface hardness analysis. F. Sterilization of specimens. G. Selection of volunteers and molding of their mouth. H. Use of the intraoral appliance for 30 minutes – Formation of the initial acquired pellicle. I. Treatment of specimens. J. Use of the intraoral appliance for 1 hour – Obtaining the modified pellicle. K. Erosive challenge (30 sec) under constant agitation. L. Final surface hardness analysis.

The experimental design is shown in Figure 1. The AEP was formed *in situ* by 5 volunteers, who wore a palatal intraoral device with the enamel blocks for 30 minutes. The sample size was calculated considering a minimally detectable difference of 15% hardness loss, standard deviation of 7.5%,^6^ 14 independent groups, α error of 5% and β error of 20%, totalling 10 enamel blocks per group. The enamel blocks were treated *in vitro* according to the groups (n = 10): C+ - SnCl_2_/NaF/AmF-containing solution (Elmex^®^ Erosion Protection Dental Rinse/EP – CP GABA GmbH; Hamburg, Germany) (positive control); C- - deionized water (negative control); O - palm oil (Kidendê – Dendê Light Indústria de Produtos Alimentícios Ltda, Valença, BA, Brazil); P6.5 - 6.5% proanthocyanidin (95% dry extract Vitis vinifera – Florien Fitoativos, Piracicaba, Brazil); P2 - 2% proanthocyanidin (95% dry extract Vitis vinifera – Florien Fitoativos, Piracicaba, SP, Brazil); E- Vitamin E (97.8% oily tocopherol acetate – Lepuge Insumos Farmacêuticos Eireli, São Bernardo do Campo, Brazil); OP6.5 - palm oil + 6.5% proanthocyanidin; P6.5O - 6.5% proanthocyanidin + palm oil; OP2 - palm oil + 2% proanthocyanidin; P2O - 2% proanthocyanidin + palm oil; EP6.5 - Vitamin E + 6.5% proanthocyanidin; P6.5E - 6.5% proanthocyanidin + Vitamin E; EP2 - Vitamin E + 2% proanthocyanidin; P2E - 2% proanthocyanidin + Vitamin E. The order in which the groups containing combinations of agents are described corresponds to the order in which the treatments were applied. All solutions were administered using a dropper (5 drops, ensuring complete coverage of the entire enamel surface) for a duration of 30 seconds, consistent with the recommended application time for Elmex^®^ Erosion Protection. After the treatment, the palatal device was tilted at a 90-degree angle to facilitate the drainage of any excess, and subsequently, the device was reinserted into the oral cavity for 1 hour for additional pellicle formation. Then, the enamel blocks were immersed in citric acid (0.5%, pH 2.5) for 30 seconds and then washed with deionized water. The response variable was percentage of hardness loss.

### Enamel blocks preparation

Enamel blocks (4x4x3 mm^3^, n = 200) were obtained from labial surfaces of bovine incisor crowns, which were cut with two diamond discs (Extec Cor.; Enfield, United States) separated by a 4 mm-thick spacer using an IsoMet^®^ low speed saw cutting machine (Buehler Ltd.; Lake Bluff, United States). Then, the surface of enamel blocks was smoothed with water-cooled silicon carbide discs (320, 600, and 1200 grade papers; Buehler Ltd.; Lake Bluff, United States), and wet polished with felt paper and diamond spray (1 µm; Buehler Ltd.; Lake Bluff, United States). The initial surface hardness analysis was determined based on the mean value of 5 indentations made 100 µm away from each other with a Knoop diamond indenter (25g for 10 seconds) (HMV-2, Shimadzu Corp., Kyoto, Japan). Specimens that showed values 10% higher or lower than the mean were excluded to avoid bias regarding initial enamel condition, thus 140 enamel blocks were selected. The blocks were randomized among the 14 groups and the five volunteers (position of the block in the intraoral appliance). Subsequently, they were sterilized by ethylene oxide gas exposure.

### *In situ* phase –AEP formation and modulation

Five adult volunteers (aged 20 to 25 years) participated in the study after undergoing examination and meeting the following inclusion criteria: physiological salivary parameters (stimulated flow > 1 mL/min; unstimulated flow >0.1 mL/min; neutral pH 7.0–7.5), and good oral health (no dental erosion, periodontitis, or untreated carious lesions). Exclusion criteria were the presence of systemic diseases, undergoing radiotherapy or chemotherapy, use of medications affecting salivary characteristics (such as antidepressants, narcotics, diuretics, or antihistamines), history of gastroesophageal reflux or frequent regurgitation or vomiting, smoking, pregnancy or breastfeeding, engagement in sports involving exposure to low-pH treated water (e.g., swimming), or topical fluoride application within the last two months. Intraoral palatal appliances were made in acrylic resin containing two sites (6x6x3 mm^3^) for fixing two enamel blocks.

The appliance was inserted into the oral cavity in the morning (one hour after oral hygiene) and was left in place for 30 minutes (8:00 to 8:30 AM). Then, the appliance was removed, and the treatment of the blocks was conducted. Vitamin E and palm oil were used in their original formulation. The proanthocyanidin solution was manipulated at a concentration of 6.5 and 2%. Considering that polyphenol, such as proanthocyanidin, has the ability to precipitate proteins from the pellicle,^15^ after treating the blocks, the palatal intraoral device was reinserted into the oral cavity for another 1 hour.

### *In vitro* phase – treatment and acid exposure

In the isolated solution groups, treatment was administered by applying 5 drops (0.25 mL) of the respective solution directly onto the enamel surface. For the groups involving combined substances, 5 drops (0.25 mL) of the first solution were applied, immediately followed by 5 drops (0.25 mL) of the second solution, in accordance with the predetermined application sequence for each study group. No removal or rinsing of the initial solution was performed prior to the application of the second solution. In all experimental groups, whether solutions were applied individually or in combination, they were left in contact with the enamel surface for 30 seconds. The treatment was carried out on the enamel blocks inserted in the appliance to avoid any damage to the previously formed AEP. After the 30-second treatment, the palatal device was tilted at a 90-degree angle to facilitate the drainage of any excess, and it was subsequently reinserted into the oral cavity for 1 hour, for additional AEP formation.^15,16^ Demineralization was performed *in vitro* by immersing the enamel blocks in 17.6 mL (1.1 mL/mm^2^ - per block) of 0.5% citric acid (pH 2.5) under constant agitation for 30 seconds^6,8,11^. Then, each enamel block was washed with deionized water for 30 seconds to stop the demineralization process.

### Surface hardness assessment

After the erosive attack, for the analysis of final surface hardness, the same specifications of the initial reading (25 g for 10 s) (HMV-2, Shimadzu Corp., Kioto, Japan) was adopted. Five indentations were made, with a distance of 100 μm between them and 100 μm from the initial indentation and measured. The average of the indentations (final hardness after demineralization) was used to evaluate the percentage of surface hardness loss (%SHL) according to the following formula: [(initial hardness – final hardness) /(initial hardness)] X 100).

### Statistical analysis

Statistical analysis was performed with STATISTICA software system 11.0 (Stat Soft Inc.). Assumptions of equality of variances and normal distribution of errors were verified. Since the assumptions were satisfied, data were analyzed by ANOVA followed by the Fisher’s LSD test. The significance level was set at 5%.

## Results

**Figure 2:**
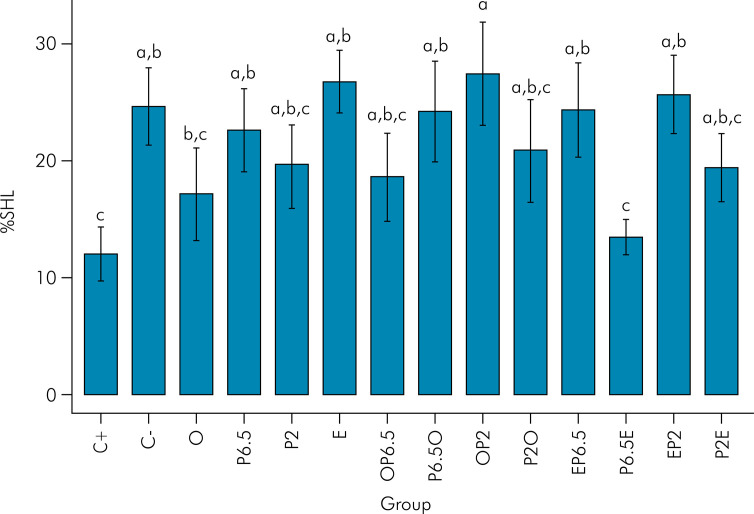
Mean and standard deviation (SD) of percentage surface hardness loss (%SHL) according to treatment groups.

The results are described in the Figure 2. The positive control group (C+ Elmex Erosion Protection^®^) and proanthocyanidin 6.5% + vitamin E (P6.5E) showed a lower percentage of hardness loss compared to the negative control group (C- deionized water) (p-values: p=0.009 and p = 0.018, respectively).

Different letters indicate a statistically significant difference between groups (ANOVA and Fisher’s LSD test p = 0.04).

O - palm oil, P2-proanthocyanidin 2%, OP6.5-palm oil + proanthocyanidin 6.5%, P2O-proanthocyanidin 2% + palm oil, P2E- proanthocyanidin 2% + vitamin E showed values of percentage of hardness loss similar to the positive control (p-values: O – p = 0.251; P2 – p = 0.118; OP6.5 - p = 0.150; P2O - p = 0.066; P2E – p = 0.119). However, these groups were also statistically similar to the negative control group (p-values: O- p = 0.122; P2 – p = 0.333; OP6.5 – p = 0.213 ; P2O – p = 0.448; P2E: p = 0.293).

P6.5 - proanthocyanidin 6.5%, E – Vitamin E, P6.5O – proanthocyanidin 6.5% + palm oil, OP2 – palm oil + proanthocyanidin 2%, EP6.5 – Vitamin E + Proanthocyanidin 6.5% and EP2 – Vitamin E + Proanthocyanidin were only similar to C- (p-values: P6.5 – p = 0.747; E – p = 0.628; P6.5O – p = 0.931; OP2 – p = 0.586; EP6.5 – p = 0.953; EP2 – p = 0.833).

## Discussion

This study assessed the protective effect of proanthocyanidin at two concentrations (2% and 6.5%) and palm oil or vitamin E, applied alone or combined, against a single short erosive challenge *in vitro* on the AEP formed *in situ*. Hardness analysis was employed, as it is a validated method for assessing the early stages of enamel dissolution and an indicator of surface demineralization. At this stage, there is no wear of the enamel, but rather softening of the dental structure.^17^ The substances that resulted in similar %SHL compared to the positive control (Elmex Erosion Protection^®^) were 2% proanthocyanidin (P2), palm oil (O), 2% proanthocyanidin + palm oil (P2O), 6.5% proanthocyanidin + vitamin E (P6.5E), 2% proanthocyanidin + vitamin E (P2E); palm oil + 6.5% proanthocyanidin (OP6.5). However, it is important to note that only the Elmex^®^ Erosion Protection (C+) and 6.5% proanthocyanidin + vitamin E (P6.5E) showed a statistically significance difference compared to the negative control. Based on the results obtained, the null hypotheses H1 and H2 were rejected.

The Elmex^®^ Erosion Protection mouthwash (a solution containing stannous chloride, amine fluoride, and sodium fluoride) has been regarded as the gold standard in preventing dental erosion^7,18^ and served as the positive control in our study. As expected, the solution resulted in a small percentage of hardness loss (around 11%). The protective effect of SnF is attributed to the incorporation of fluoride and stannous ions into the enamel, and also to the modification of the AEP^16^. Fluoride can modulate the composition of the pellicle by increasing statherin and statin concentrations.^16,19^ This modified pellicle offers enhanced protection of the tooth structure against demineralization and can even penetrate demineralized areas after acid challenges.^16,20^ In addition, stannous ions can interact with albumin,^20^ resulting in increased electron density in the basal layer of the AEP, which can remain relatively unaffected after an erosive challenge.^16,20,21^

Proanthocyanidin (6.5%) exhibited similar outcomes to both 2% proanthocyanidin and the negative control (deionized water). This contrasts with the findings of Boteon et al.,^11^ who observed that 6.5% proanthocyanidin interacted with the previously formed in situ AEP and effectively prevented enamel erosion. Discrepancies in the study protocols, including variations in AEP formation and solution application times, may account for these conflicting results. In our study, proanthocyanidin solutions were applied to the enamel for 30 seconds following AEP formation over 30 minutes, followed by an additional one-hour pellicle formation^15,16^. In contrast, Boteon et al.^11^ applied proanthocyanidin gel for 1 minute after a two-hour AEP formation period.

Proanthocyanidin is a very pigmented agent, which means that its application can cause discoloration of the tooth structure. Thus, this study proposed a reduction in its concentration in order to diminish this undesirable effect. Liu and Wang^22^ observed that the optimal concentration of proanthocyanidin in protecting dentin collagen (a type of proline-rich protein) against enzymatic digestion was around 2% or above. Therefore, the present study also investigated the effectiveness of 2%proanthocyanidin in enamel erosion and found a similar effect to that of the positive control. The protective effect of proanthocyanidin has been attributed to pellicle maturation and/or thickening through proanthocyanidin-protein aggregates, as proanthocyanidins might induce precipitation and aggregation of salivary proteins^4,14^ such as proline-rich proteins (PRPs) and histatins.^13,14^ PRPs have the ability to form resistant hydrogen bonds with proanthocyanidin and this proanthocyanidin-protein aggregate remains stable under acidic conditions.^11,13,14^

When evaluating the proanthocyanidin associations, a similar performance to the positive control was found with the 2% concentration applied before palm oil or vitamin E (P2O and P2E), and with the 6.5% concentration applied before vitamin E (P6.5E). These results indicate that proanthocyanidin has a preventive effect when applied before oily agents.

Isolated palm oil (O) also showed similar results to the positive control, reaffirming its preventive effect found in previous studies.^6-8^ The ability of tocotrienols to diffuse into the lipid layer of the cell membrane has been attributed to the antioxidant action of the oil.^9^ Hence, it was hypnotized that palm oil has the capability to permeate the lipids within the basal layer of the acquired enamel pellicle (AEP), thereby delaying its disintegration.^7,8^

Palm oil + 6.5% proanthocyanidin (OP6.5) and 2% proanthocyanidin + palm oil (P2O) exhibited outcomes comparable to the positive control. The interaction between polyphenols and lipids has been investigated, mainly through emulsification methods.^22^ Therefore, it would be interesting to investigate whether the emulsion of proanthocyanidins and palm oil interaction with AEP can contribute to the protection ability of this combination against erosive challenges.

In contrast to the results reported by Rios et al.,^8^ who observed a preventive effect of vitamin E, our study did not find an effect. Vitamin E is a fat-soluble antioxidant derived from plants consisting of four tocopherols (α, β, δ, and γ) and four tocotrienols (α, β, δ, and γ).^24^ The protective effect of vitamin E has been attributed to three mechanisms.^8^ The first is its ability to stabilize the lipid layer due to the formation of a molecular complex with the fatty acids of the pellicle,^25^ thus stabilizing the acquired pellicle. The second mechanism is through its antioxidant property,^26^ which may occur due to the diffusion capacity of tocopherol in cell membranes,^8^ allowing vitamin E to diffuse to the basal layer of the pellicle, enabling mechanisms 1 and 3. The third potential mechanism of action involves the interaction between vitamin E and specific acquired pellicle proteins.^8^ The contradicting results may be attributed to variations in the study protocols. In Rios et al.,^8^ the AEP was formed *in situ* using intraoral appliances for a two-hour period, then the specimens were treated and submitted to erosive cycling. In the present study, the AEP was formed for thirty minutes. Subsequently, the specimens were treated, and the appliances were reinserted into the mouth of the volunteers for an additional hour to modify the AEP.^15,16^ We can speculate that the AEP formation model used in this study might have affected the retention of vitamin E on the AEP, compromising their interaction. Thus, it would be interesting to investigate the effect of vitamin E with different methods of AEP formation and to carry out *in situ* studies, which can simulate the biological conditions of the oral cavity.

While isolated vitamin E did not yield favourable results in the present study, positive outcomes were observed when vitamin E was applied following concentrations of 2% or 6.5% proanthocyanidin. This may be attributed to the interaction between proanthocyanidin and lipids^23^ of the AEP and the oily vitamin E. As isolated vitamin E did not produce positive results, we hypothesize that its combination with proanthocyanidin may enhance the protective effect of oily vitamin E through an interaction with lipids. From this perspective, proanthocyanidin might have the ability to bind to AEP lipids and potentially exert simultaneous effects with vitamin E.

The interaction between proanthocyanidin and vitamin E may also have occurred due to the ability of both to bind to specific AEP proteins. Another possible protection mechanism of vitamin E against enamel erosion is its ability to interact with AEP proteins, particularly Afamin.^8^ Afamin is the alternative name for alpha-albumin^27^. It is a specific protein of the serum albumin family^28^ commonly found in the AEP^29^ that can bind vitamin E^30^. Proanthocyanidin, in turn, can bind to serum albumin.^13,31^ Li et al.^31^ evaluated the interaction between human serum albumin (HSA) with α-tocopherol (the most biologically active form of Vitamin E) and proanthocyanidins. The study revealed that these agents bind to different sites on HSA, proanthocyanidin binding to site I and α-tocopherol to site II. Consequently, when applied simultaneously, proanthocyanidin and α-tocopherol do not interfere with each other’s interaction with HSA, as they do not compete for the same binding site.^31^ Therefore, proanthocyanidin and α-tocopherol can induce conformational and microenvironment changes in HSA in a balanced manner.^31^ We can assume that the ability of proanthocyanidin and vitamin E to bind to albumin can be considered a factor of interaction between these agents with AEP, potentially contributing to protect against erosion. This interaction mechanism may also explain the observed optimal protective potential of 6.5% proanthocyanidin when combined with vitamin E (P6.5E), which was the only combination investigated that was similar to the positive control and different from the negative control. Nevertheless, this interaction should be investigated to validate its viability as a mechanism of action,^8^ possibly through proteomic analysis of the AEP. Additionally, further studies should explore the impact of this combination over prolonged erosion periods coupled with abrasion and in situ investigations that simulate the biological conditions of the oral cavity should be conducted. This association (P6.5E) holds the potential to serve as the foundation for the development of an effective and cost-efficient anti-erosion product in the future.

## Conclusion

Based on our study design, palm oil, 2% proanthocyanidin, and their associations with either vitamin E or with each other (OP6.5, P2O, P2E) exhibited a protective effect against initial enamel erosion, showing an intermediate performance between the negative and positive controls. In contrast, the combination of 6.5% proanthocyanidin and vitamin E (P6.5E) stood out as the most effective strategy, with results close to the positive control. These findings highlight the potential of combining natural agents for enhanced enamel protection.

## Data Availability

Data is available on demand from the reviewers.
